# Transcriptional and epigenetic dynamics of sex determining gene *PdFERR* and MADS-box related genes during flower development in *Populus deltoides*

**DOI:** 10.3389/fpls.2025.1582915

**Published:** 2025-05-13

**Authors:** Yu-Jie He, Cong Chen, Ying Guo, Jian Tang, Jie He, Li-Na Mei, Yousry A. El-Kassaby, Huaitong Wu, Tongming Yin, Liang-Jiao Xue

**Affiliations:** ^1^ State Key Laboratory of Tree Genetics and Breeding, Co‐Innovation Center for Sustainable Forestry in Southern China, Key Laboratory of Forest Genetics & Biotechnology of Ministry of Education, Nanjing Forestry University, Nanjing, China; ^2^ Department of Forest and Conservation Sciences, Faculty of Forestry, The University of British Columbia, Vancouver, BC, Canada

**Keywords:** poplar, flower development, sex determination, MADS-box genes, gene regulatory network

## Abstract

Manipulation of genes controlling sex differentiation, flower development, and flowering in poplar is pivotal to shortening the juvenile phase for a speed breeding system or eliminate flowering to reduce the dispersions of polluting pollens and hairy seeds. The sex-determining gene (*PtARR17*/*PdFERR*) and some core transcriptional regulators, such as the MADS-box gene *AGMOUS*, have been identified in *Populus*. However, the interactions among them have not been explored well. Here, we integrated RNA-seq, small RNA-seq, and Bisulfite-seq to characterize the dynamics of regulatory genes at multiple levels. Ninety-six MADS-box genes were identified, which can be grouped into 6 clusters based on expression level. The E-class genes exhibited diverse expression patterns, suggesting differentiated regulatory roles. Through deep sequencing, 236 miRNAs targeting more than 4500 genes were annotated. Eight MADS-box genes were predicted as direct targets of miRNAs. At the genome level, DNA methylation at stage T2 is higher than in the later stages. More than 10K genes were differentially methylated between female and male flower buds, indicating the significant regulatory roles of DNA methylation in flower differentiation. The MADS-box-centered regulatory network consists of co-expressed transcription factors, and miRNA genes were constructed. The correlations between *PdFERR* and transcription factors, including MADS-box genes and other environment-responsive genes, provide clues to understand the labile sex expression. Our study provides candidate genes for engineering of flower development process for trait improvement.

## 1 Introduction

*Populus* is a pivotal model species for studying tree biology and forest genetics due to its unique combination of wood production, ecological relevance, genomic resources, and adaptability to fundamental experimental research (Douglas, 2017). The life span of poplar trees is 100 to 200 years, and the juvenile phase is 7 to 10 years (Hsu et al., 2006). The studies of regulatory mechanisms in flower development would advance the breeding of elite poplar cultivars for diverse objectives. The shortening of the juvenile phase can facilitate the development of a speed breeding system (fast-track breeding approach) for poplar (Wenzel et al., 2013). The delaying or elimination of flowering can relocate more carbohydrates from photosynthesis to vegetative growth. The elimination of flowering can also prevent the spread of pollens and catkins, which are critical for controlling air pollution and the risks associated with the spread of transgenic pollens (Azeez and Busov, 2021). As a genus with predominantly dioecious species, the development processes of flowers are closely linked with sex-determination mechanisms (Gladysh et al., 2024). The variations in expression levels of genes in flower development can be induced by dynamics of sex-determining genes, which in turn results in the labile expression of sex traits (Käfer et al., 2022; Mao et al., 2023; Blake-Mahmud and Struwe, 2018).

The flower development is controlled by genes of the ABC model, which is the foundational framework to elucidate how floral organs are specified in four whorls including sepals, petals, stamens, and carpels (Soltis et al., 2007). In the model, A-class genes alone specify sepal identity, A- and B-class genes together regulate petal formation, B- and C-class genes together govern stamen development, and C-class genes alone control carpel identity (Soltis et al., 2007). Subsequently, the D-class genes *FLORAL BINDING PROTEIN7* (*FBP7*) and *FBP11* were identified in Petunia hybrida and shown to play an important role in ovule development (Angenent et al., 1995). Additionally, *SEPALLATA1* genes (*SEP1*, *SEP2*, *SEP3*, and *SEP4*) function as E-class genes in Arabidopsis thaliana, being expressed in all four floral whorls and contributing to the regulation of floral organ identity (Pelaz et al., 2000). The E-class genes are functionally redundant with ABC homeotic genes in specifying the identity of all types of floral organ types (Cheng et al., 2019). As a result, the original ABC model was expanded to the ABCDE model (Soltis et al., 2007). With the exception of *AP2*, all ABCDE genes encode MIKC-type MADS-domain proteins (Irish, 2010). The identifications of MADS-box genes and their transcriptional and post-transcriptional regulators could provide gene targets for perturbation of flower development in poplar.

Diverse environmental and developmental signals are integrated through regulators to tune the expression of MADS-box genes (Chen et al., 2018). *FLOWERING LOCUS C* (*FLC*) represses the expression of *SUPPRESSOR OF OVEREXPRESSION OF CO 1* (*SOC1*) to integrate signals from vernalization, long-day, and gibberellin (GA)-dependent flowering pathways. Both *FLC* and *SOC1* are also members of the MADS-box gene family (Moon et al., 2003). The floral homeotic genes are also regulated by upstream transcriptional regulators, such as *LEAFY* (*LFY*) (Weigel et al., 1992). In addition to transcriptional regulation, microRNAs (miRNAs) also play roles in the flower development pathways. miR156 and miR172 are involved in age-dependent pathways controlling flowering time (Nag and Jack, 2010). miR172 targets *APETALA2* (*AP2*), an A-class gene, to establish the boundary between the perianth and reproductive organs (Wollmann et al., 2010). miR319 and miR164 target *TCP* and *CUC* transcriptional factor families, respectively, to control petal development (Nag et al., 2009; González-Carranza et al., 2017). The functions of miRNAs targeting genes in the ABCDE model increased the complexity of gene regulatory networks in flower development.

DNA methylation, the addition of methyl groups to cytosine residues, plays a significant roles in regulating flower development by dynamically controlling gene expression (Shi et al., 2022). The dynamics of DNA methylation were also observed at the whole genome level during flower development in diverse plant species (Liang et al., 2014; Yang et al., 2015). The expression of central flowering repressor *FLC* is regulated through DNA and histone methylation (Ito, 2012). In rose, the DNA hypermethylation of *AGAMOUS* (*AG*, C-class) is induced by low temperature, which results in the attenuated expression level and increased petal number (Ma et al., 2015). The reduced DNA methylation in *Arabidopsis* results in the ectopic expression of *APETALA3* in leaf tissue and flower homeotic transformations (Finnegan et al., 1996). In poplar, the sex-determining gene *PtARR17* in *Populus* tremula and its homolog *PdFERR* in *Populus deltoides* (*P. deltoides*) is hypermethylated at the promoter region and the first exon in male plants. The reduced methylation levels in female plants result in the expression of *PtARR17*/*PdFERR* and promote the development of female flower buds (Müller et al., 2020; Xue et al., 2020).

In this study, we applied RNA-seq, small RNA-seq, and Bisulfite-seq techniques to characterize the dynamics of regulatory genes in transcriptional, post-transcriptional, and DNA methylation levels. Genes predominantly expressed in poplar flower buds and genes differentially expressed during flower development were identified. The expression patterns of MADS-box genes were explored and applied to identify co-expressed transcriptional factors. The dynamics in DNA methylation was analyzed at the whole genome level with a focus on the identified MADS-box genes and their regulators. The identified regulators of other MADS-boxes genes are considered as “driver genes”, which can serve as candidates for engineering of flower development process in poplar. The epigenetic modifications and interactions of regulatory genes would help to understand the cross-talks between flower development pathways and signaling pathways in response to diverse environmental factors.

## 2 Materials and methods

### 2.1 Plant materials

Plant samples of *P. deltoides* were collected from poplar trees planted on the Xinzhuang Campus of Nanjing Forestry University, Jiangsu, China (latitude: 32.078682 °N, longitude: 118.816826 °E). The poplar trees are about 20-meters in height and planted under un-controlled environments. The flower buds of female and male trees were harvested at five stages: T1 (18th June 2018), T2 (3rd July 2018), T3 (3rd August 2018), T4 (1st December 2018), and T5 (15th January 2019). In addition to flower buds, we also collected vegetative tissues, including root, stem, and leaf tissues at stage T1. For each sample, we harvested three biological replicates. The sample information the is shown in **Supplementary Table S1**, together with the temperature and weather of sampling date.

The morphological characteristics of flower buds have been detailed in a publication from our research group (Lu et al., 2022). In summary, at stages T1 and T2, the flower buds undergo bract differentiation (female) and floret primordium initiation (male), with the bracts differentiating into florets. At stage T3, the flower buds reach the stage of gynoecium/androecium differentiation; fully developed pistils are present in female flower buds, while pollen sacs form in male flower buds. During stages T4 and T5, floral buds enter dormancy phases where the development of pistil and stamen primordia is temporarily halted.

To investigate differentially expressed protein-coding genes and miRNAs during flower development, we selected flower bud samples from stages T1, T3, and T4 for RNA sequencing as well as small RNA sequencing. For Bisulfite sequencing purposes, a relatively large quantity of samples is required; thus, samples from later stages (T2, T4, and T5) were collected for library construction.

### 2.2 RNA sequencing and data analysis

Total RNA was extracted using a Trizol reagent kit (Invitrogen, Carlsbad, CA, USA) according to the manufacturer’s protocol. After the examination of quality and concentration with NanoPhotometer^®^ (Implen, CA, USA) and RNA 6000 Nano Kit (Agilent Technologies, CA, USA), the RNA samples were applied for cDNA libraries construction and sequenced on the NovaSeq™ 6000 platform. Paired-end reads of 150 bases were sequenced for each library.

To obtain high-quality reads, adaptors of raw reads were removed by Trimmomatic (version 0.39) (Bolger et al., 2014). To quantify mRNA expression, the TPM (Transcripts per million) value of each transcription region was calculated using RSEM (version 1.3.3) (Li and Dewey, 2011). Principal component analysis (PCA) was performed using the expression levels of all samples. Differentially expressed genes (DEGs) were identified using DESeq2 (Love et al., 2014) and Analysis of Variance (ANOVA) with a threshold of FDR < 0.05 and |logFC| > 1.5. We performed three sets of transcriptome analyses (Analysis I, Analysis II, Analysis III) using samples of male and female flower buds collected at T1, T3, and T4, as well as samples of other tissues (roots, stems, and leaves) (**Supplementary Table S2**). Firstly, flower bud samples were analyzed against other tissues using the DEseq2 software package for genes that were highly expressed in the flower buds (Analysis I). Secondly, we performed ANOVA on female (male) flower bud samples at the T1, T3, and T4 stages to obtain genes that were differentially expressed in the flower buds (Analysis II). Finally, differential expression analyses were performed between female and male flower bud samples at the T1, T3, and T4 stages to screen for genes differentially expressed between females and males (Analysis III).

### 2.3 Small RNA sequencing

Consistent with RNA-seq, sRNA libraries of male and female buds from the stages T1, T3, and T4 were constructed through TruSeq Small RNA Sample Prep Kits and sequenced using the Illumina Hiseq2000/2500 sequencer (50-bp single-end reads). The small RNA reads were processed using sRNAminer (Li et al., 2024) software to identify known and novel miRNAs and obtain the expression levels. Differential expression analysis of miRNAs was performed using the limma (Ritchie et al., 2015) package (Analysis IV). MiRNAs and transcript sequences were submitted to the online tool psRNATarget to conduct target gene prediction with default setting (maximum cutoff of score = 3.5) (Dai et al., 2018).

### 2.4 Bisulfite sequencing and methylation analysis

The sequencing libraries of male (MMT2, MMT4, and MMT5) and female (MFT2, MFT4, and MFT5) bud tissue samples from three stages (T2, T4, and T5) were constructed with the EZ DNA Methylation-Gold™ Kit (D5006). The insertion size and effective concentration of libraries were assessed using the Agilent 2100 Bioanalyzer and StepOnePlus™ Real-Time PCR system. The libraries were sequenced using the HiSeq X10 platform. During data analysis, the low-quality reads and adapter sequences were firstly removed by Trimmomatic (version 0.39) with default parameters. Then, filtered reads were aligned onto the *P. deltoides* reference genome using Bismark (version 0.22.3) with bowtie2 as the aligner. Methylation ratios of cytosine sites were calculated based on mapped reads as the number of C divided by C+T. DNA methylation levels of genomic regions were estimated by the ratio of methylated cytosines to all cytosines in CG, CHG, and CHH contexts of the examined regions. We applied the CGmap-Tools (version 0.1.2) (Guo et al., 2018) to calculate methylation levels of TEs and genes.

### 2.5 Identification of differentially methylated regions

To call differentially methylated regions (DMRs) between male and female samples at different stages, the genome sequences were first divided into bins of 200 bp with a sliding step size of 100 bp. The significance of differential methylation was analyzed using methylKit (version 1.24.0) (Akalin et al., 2012). Genes overlapping with one or more DMRs in the gene body and its upstream and downstream 2-kb regions were defined as differentially methylated genes (DMGs).

### 2.6 Genome-wide identification of MADS-box genes

To identify MADS-box gene family members in *P. deltoids*, we used iTAK (version 1.7) (Zheng et al., 2016) to predict transcription factors at the whole genome level. The protein sequences of the MADS-box family were extracted and combined with homologous sequences from *Arabidopsis* and rice. Phylogenetic trees were constructed using the Maximum-likelihood method with MEGA software. The categorizes of MADS-box genes in *P. deltoids* were determined based on the phylogenetic tree.

### 2.7 Construction of MADS-box centered regulatory network

The regulatory network was constructed based on gene co-expression analysis and interaction pairs between miRNAs and their targets. Genes with low expression levels (TPM <2) were removed from further analysis. The correlations of gene pairs were calculated using weighted gene co-regulatory network analysis (WGCNA). MADS-box genes of the ABCDE model and their co-related genes were extracted. miRNAs targeting genes in the co-expression network were predicted using psRNATarget. The network was constructed using CytoScape (version 3.7.1) (Smoot et al., 2011), and genes of transcription factors and protein kinases were highlighted.

## 3 Results

### 3.1 Identification of differentially expressed genes during flower bud development

Transcriptional sequencing was applied to identify key regulators of flower development. Principal component analysis (PCA) was performed to check the reproducibility of transcriptional data. The results revealed the close distances between samples of biological replicates. This clustering also indicated samples of female and male individuals are closely related, suggesting the major factors distinguishing the transcriptional data are tissue types and developmental stages (**Supplementary Figure S1**).

**Figure 1:**
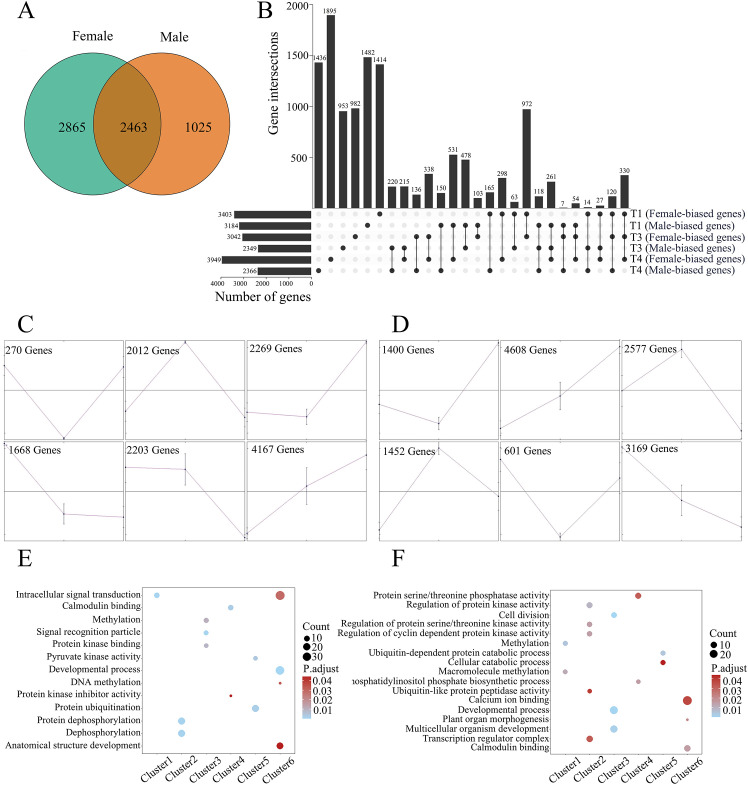
Transcriptional dynamics during the development of female and male flower buds in *Populus*. **(A)** Venn diagrams showing numbers of genes predominantly expressed in female and male flower buds. **(B)** Numbers of genes differentially expressed between female and male flower buds at T1, T3, and T4 stages. The gene numbers of different overlapping categories were also exhibited. **(C-D)** Clustering of differentially expressed genes during flower bud development for female **(C)** and male **(D)** plants, respectively. The x-axis indicates the development stages of T1, T3, and T4. **(E-F)** GO enrichment analysis of genes from six clusters for female **(E)** and male **(F)** plants.

In our analysis, we focused on three groups of genes differentially expressed in poplar flower buds. Firstly, genes predominantly expressed in flower buds were identified by comparing flower samples and vegetative samples (root, stem, and leaf) for females and males, respectively. 5,328 and 3,488 genes were highly expressed in female and male flower buds, with 2,463 genes present in both sexes (**Figure 1A**). GO enrichment analysis indicated that genes involved in RNA processing and developmental process are over-represented in genes highly expressed in both female and male flower buds (**Supplementary Figure S2**). At the gene level, many genes involved in the regulation of the development of petals, calyx, stamens, pistils, and ovules were highly expressed in female and/or male flower buds, including genes of MADS-box, WOX, and NAP families (**Supplementary Figure S3**) (Sablowski and Meyerowitz, 1998; Du et al., 2024).

In the second group, we focused on differentially expressed genes (DEGs) among the developmental stages (T1, T3, and T4) of flower buds. Through the analysis of one-way ANOVA, 12,589 and 13,808 were identified as DEGs in female and male flowers, most of which (9729 genes) were shared in two sets (**Supplementary Figure S4**). The sets of DEGs were further clustered into six clusters. As shown in **Figures 1C and 1D**, the clusters exhibiting gradually increased expression patterns are major clusters for both female (cluster 6) and male (cluster 2) flower buds. For the two major clusters, genes in intracellular signal transduction and developmental process were enriched for female flower buds, and genes in the regulation of protein kinase activity and transcription regulator complex were enriched for male flower buds (**Figures 1E, F**).

In the third group, DEGs between female and male flower buds in three developmental stages were identified. As shown in **Figure 1B**, the numbers of sex-biased DEGs were comparable at stage T1; 3,403 genes are female-biased, and 3,184 ones are male-biased. At stage T2, the difference between female-biased and male-biased genes was 693, and the number increased to 1,583 at stage T3 (**Figure 1B**). The numbers of biased genes agree with the morphological differences of flower buds. Among the common genes shared in two gene sets, 972 genes exhibited female-biased patterns in both the T1 and T3 stages and 531 genes were male-biased expressed in both the T1 and T4 stages. Three hundred thirty genes were female-biased in all three stages, whereas 120 genes were female-biased in T1 and T3 but male-biased in T4. The diverse expression patterns of genes indicated significant dynamics in transcriptional regulation during flower development.

### 3.2 Phylogenetic and expression analysis of ABCDE genes in MADS-box family

**Figure 2:**
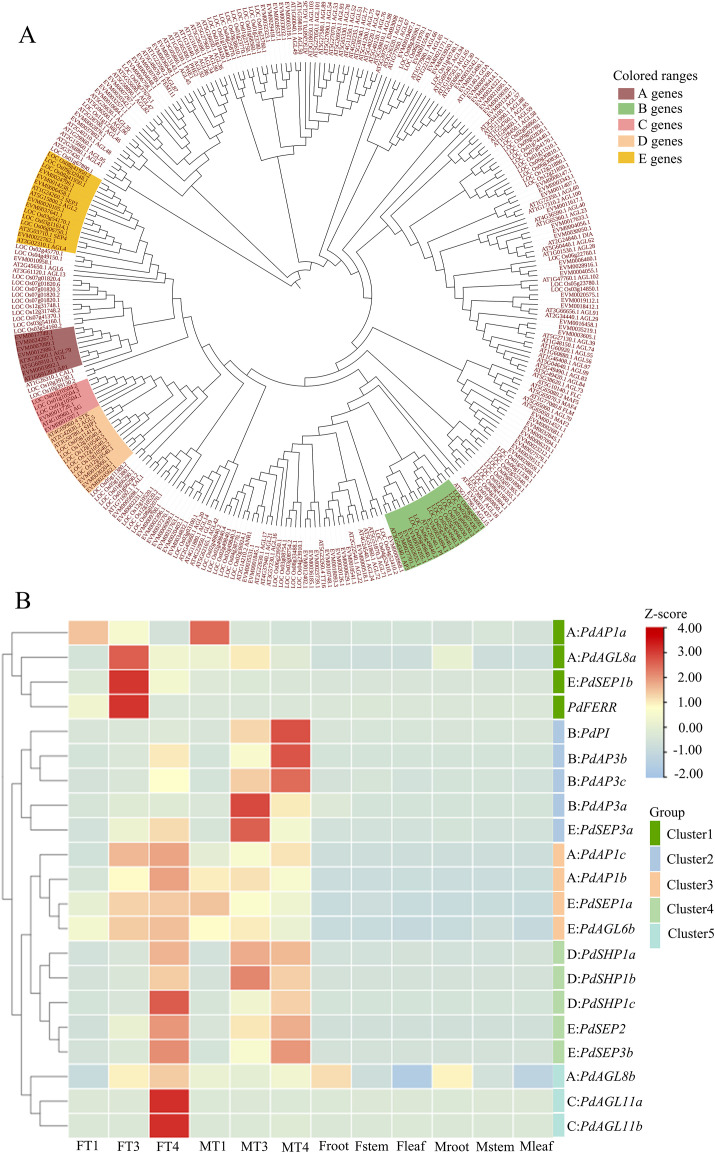
Phylogenetic tree and expression patterns of ABCDE genes. **(A)** Phylogenetic tree constructed with genes of MADS-box gene family in *P. deltoides, Arabidopsis*, and *Oryza sativa*. **(B)** Expression patterns of *PdFERR* and genes in ABCDE model in flower buds and vegetative tissues including root, stem, and leaf. F and M indicate female and male plants, respectively. The visualized data has been normalized through log_2_ (TPM+1). The letters ABCDE before the colon represent which class of ABCDE genes this gene belongs to.

Genes in the MADS-box family play pivotal roles in processes of floral organogenesis, differentiation, and morphogenesis, which include key genes in the ABCDE model of flower development. Through a similarity search of conserved protein domains, we identified 96 MADS-box gene family members (**Supplementary Table S3**, S4). To identify ABCDE genes in *P. deltoides*, phylogenetic analysis was performed using MADS-box genes of *P. deltoides*, *Arabidopsis*, and rice (**Figure 2A**). Genes of *P. deltoides* were named following genes from the same clades with *Arabidopsis* and rice. As a result, 5 A genes, 4 B genes, 2 C genes, 3 D genes, and 6 E genes were identified (**Supplementary Table S4**).

The expression patterns of genes in the MADS-box family were further characterized. The results indicated that most of them exhibited tissue-predominant expression (**Supplementary Figure S5**). As shown in **Figure 2B**, the expression patterns of ABCDE genes were grouped into 5 clusters. Genes of B-, C-, and D-class were grouped into clusters 2, 5, and 4, respectively. Among them, C genes were specifically expressed in T4 of female flower buds, B genes were predominantly expressed in male flower buds at the stages T3 and T4, and D genes were highly expressed in the T4 stage of both female and male flower buds. Genes of A- and E-class were grouped into multiple clades, suggesting functional diversity of these genes. It is noteworthy that *PdFERR* was highly expressed in female buds of stage T3 and grouped together with two A-class genes and one E-class gene (cluster 1).

### 3.3 Differentially expressed miRNAs during poplar flower development

To identify regulatory miRNAs controlling the development of flower buds, small RNA sequencing was performed for samples same as used in the RNAseq experiment. All miRNA loci were identified through genome-wide prediction. A total of 236 miRNAs were annotated, including 199 known and 37 novel miRNAs (**Supplementary Table S5**). Further, a total of 4,584 genes were predicted to be target genes of miRNAs (**Supplementary Table S6**), among which 8 MADS-box genes were predicted as targets (**Supplementary Table S7**).

**Figure 3:**
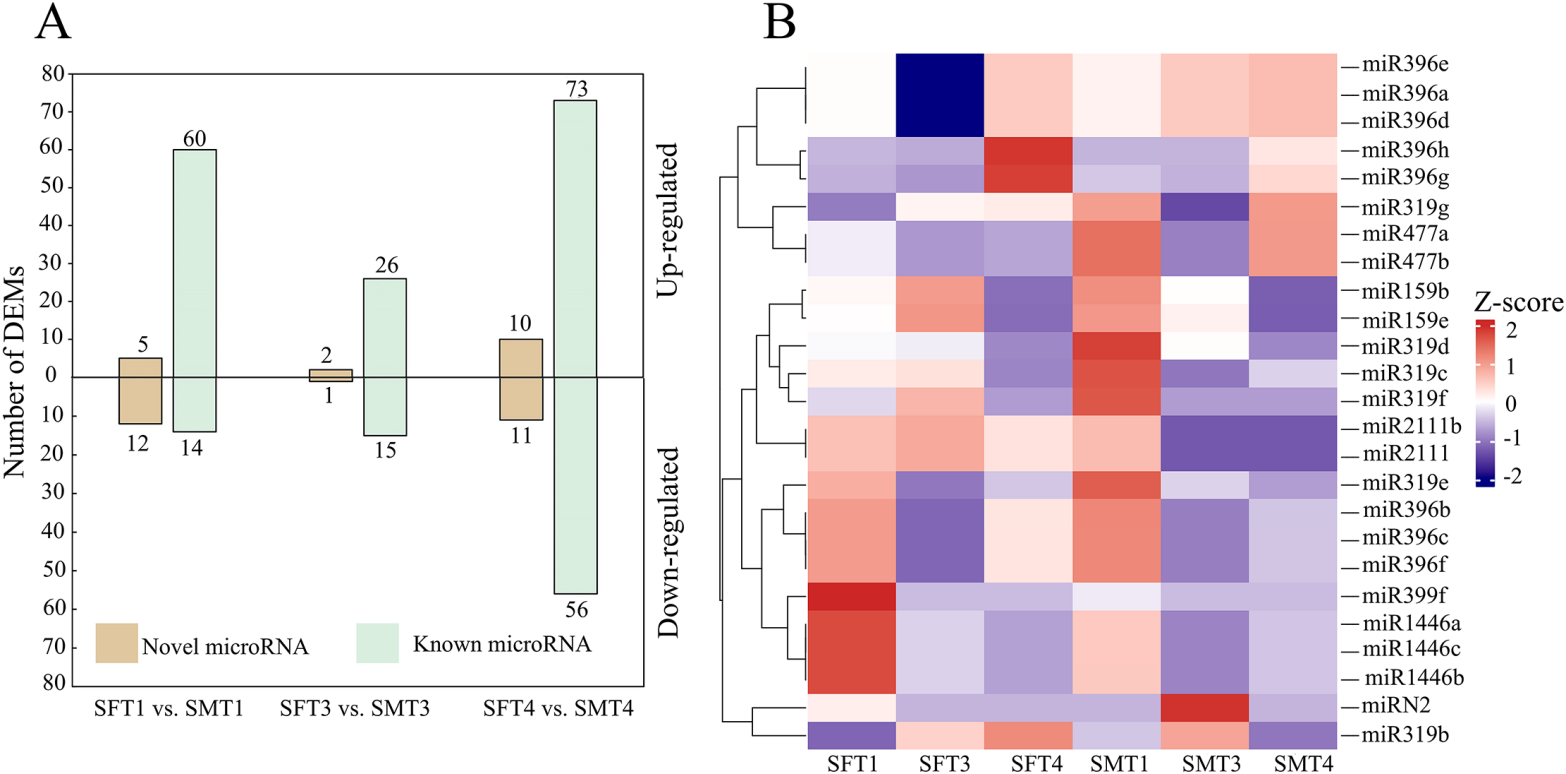
Numbers of differentially expressed miRNAs between female and male flower buds and expression patterns of miRNAs predicted to target MADS-box genes. **(A)** Numbers of novel and known miRNAs are differentially expressed at three stages. Above the x-axis: male >female, below the x-axis: male <female. **(B)** Heatmap showing the expression patterns of miRNAs targeting MADS-box genes. The expression values were normalised by each gene. S indicates small RNA, F and M indicate female and male, respectively.

We further performed differential expression analysis of miRNAs between female and male flower buds. A total of 91, 44, and 150 differentially expressed miRNAs (DEMs) were obtained for developmental stages T1, T3, and T4, respectively (**Figure 3A**). Most of these DEMs were known miRNAs, of which male-biased expressed miRNAs were higher than female-biased miRNAs (**Figure 3A**). The expression patterns of miRNAs targeting MADS-box genes were explored. The results indicated that most of these miRNAs gradually down-regulated during flower development, especially in male buds, consistent with gradually increased expression of MADS-box genes (**Figure 3B**).

### 3.4 Dynamics of DNA methylation patterns in poplar flower buds

**Figure 4:**
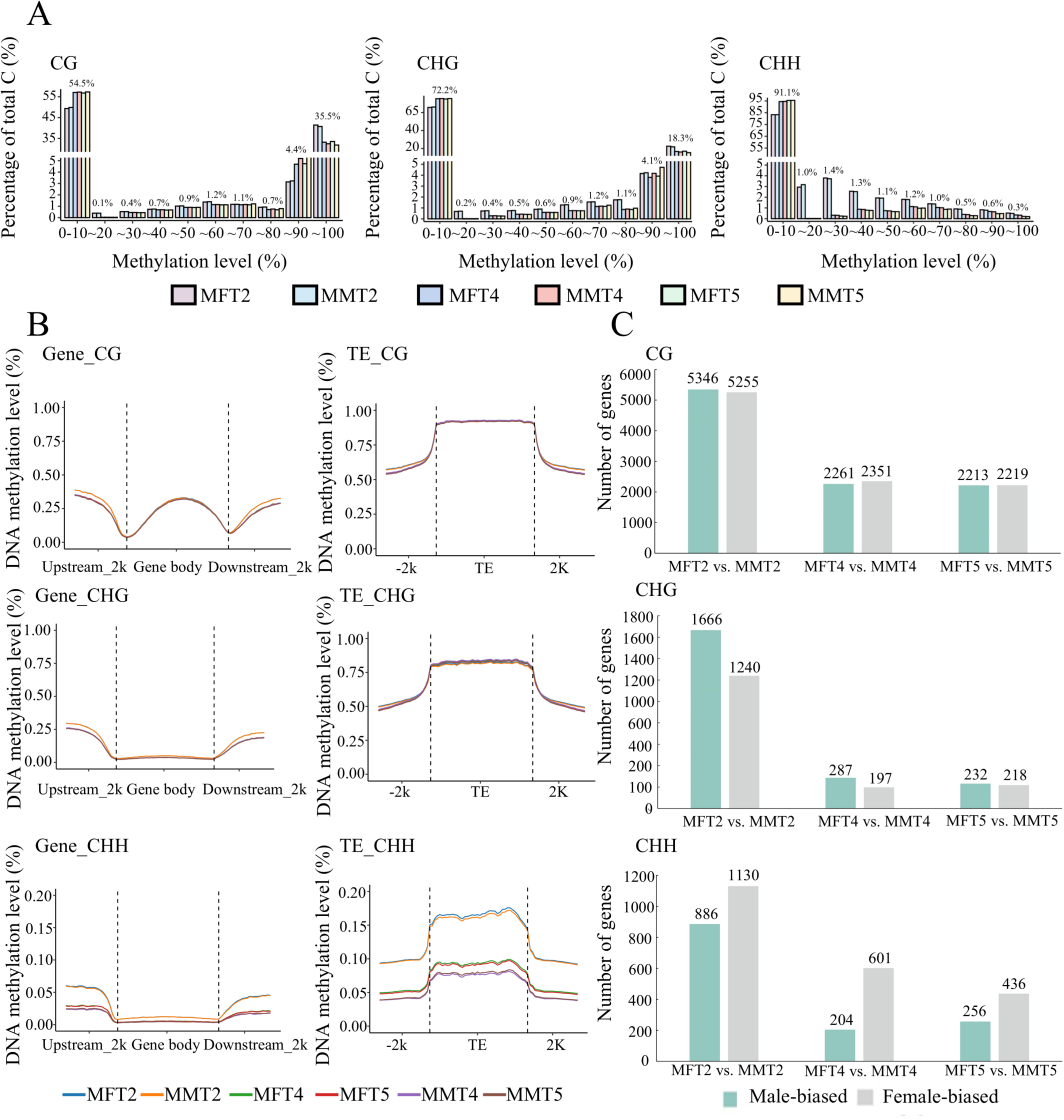
DNA methylation patterns of female and male flower buds in *P. deltoides.* **(A)** Proportional distribution of DNA methylation levels in CG, CHG, and CHH contexts in male and female flower buds at three developmental stages. **(B)** DNA methylation patterns in gene space of protein-coding genes and TEs. **(C)** Numbers of Differentially Methylated Genes (DMGs) between female and male flower buds at three stages. The first M in the sample name indicates DNA methylation samples.

During flower development, DNA methylation patterns have been reported to be associated with gene expression levels. In our analysis, approximately 41 and 24% of the CG and CHG context exhibited high methylation rates (>80%), while the rate in CHH context was 1% (**Figure 4A**). The DNA methylation levels in all three contexts were higher at T2 stage in both female and male flower buds than in samples at the stages T4 and T5 (**Figure 4A**). In gene space, the average DNA methylation profiles indicated that the gene body methylation decreased at transcription start sites (TSSs) and transcription end sites (TESs) in the CG context. Meanwhile, relative depletion of DNA methylation percentage was observed in genic regions compared with the surrounding sequences in both CHG and CHH contexts (**Figure 4B**). The methylation levels of transposable element (TE) bodies were higher than their surrounding sequences in all three contexts (**Figure 4B**). The distribution patterns of DNA methylation in CG and CHG contexts were similar in all samples of female and male flower buds, whereas the methylation levels in samples at stage T2 were higher than in samples of T4 and T5 in CHH context (**Figure 4B**).

We further identified differentially methylated genes (DMGs) between male and female flowers at three development stages. In all three contexts, the numbers of DMGs are higher in T2 than in other developmental stages (**Figure 4C**). In the CG context, the numbers of male and female-biased DMGs are similar. In the CHG context, more male-biased DMGs were detected than female-biased DMGs, and the pattern was reversed in the CHH context (**Figure 4C**). Among the DMGs, the sex-determining gene *PdFERR* exhibited a male-biased pattern in CG and CHG contexts. A total of 28 MADS-box genes were differentially methylated between male and female samples (**Supplementary Table S8**).

### 3.5 Construction of regulatory gene network in flower development

**Figure 5:**
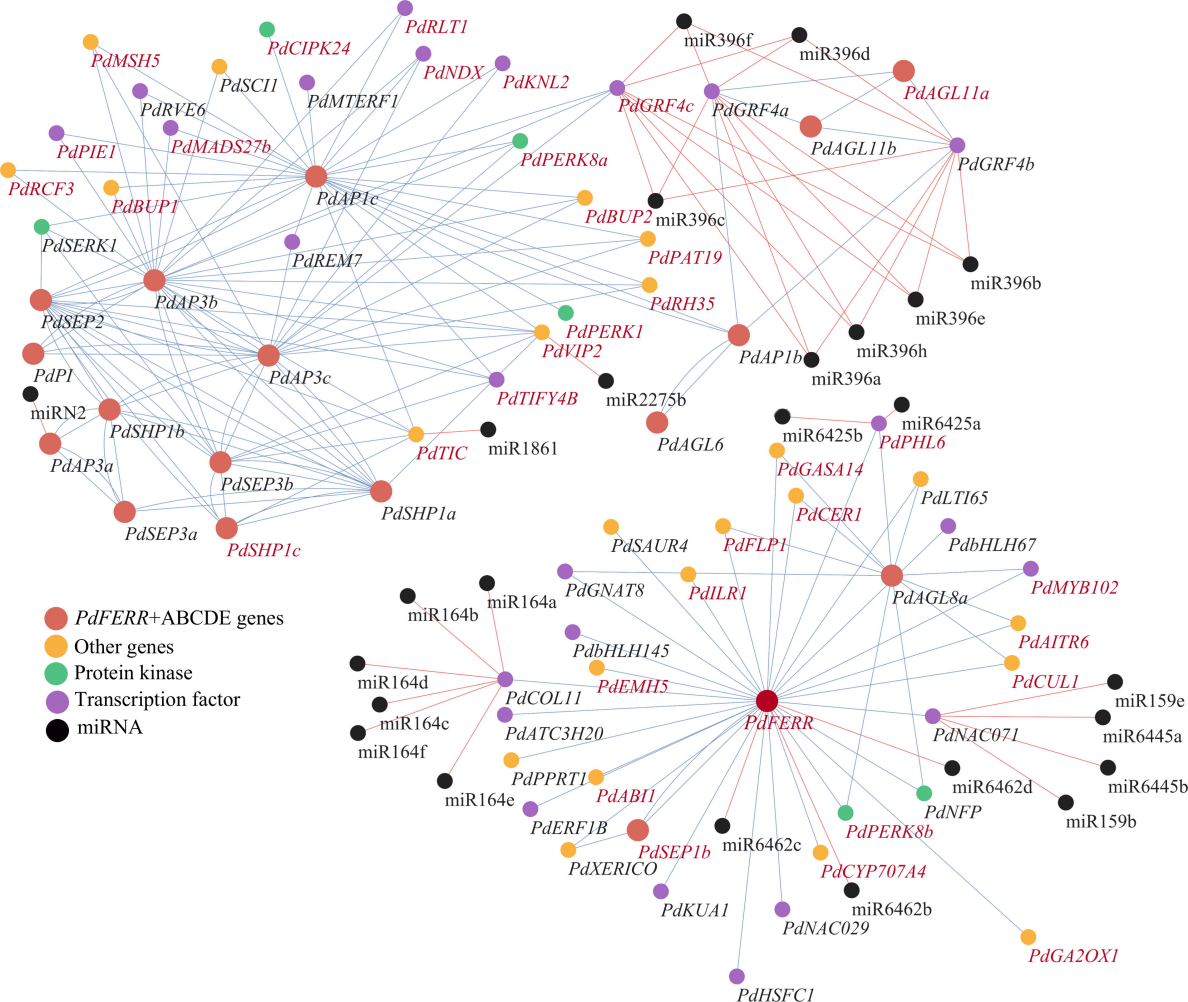
Regulatory network of ABCDE genes and the associated miRNA and transcription factor genes. ABCDE genes and *PdFERR* are indicated in red; miRNAs are indicated in black; protein kinases, transcription factors, and other protein-coding genes are indicated in green, purple, and orange, respectively. The genes highlighted in red represent differentially methylated genes (DMGs). Blue and red lines indicate positive and negative correlations, respectively.

To explore the interactions of key regulators in flower development, a regulatory network was constructed for MADS-box genes and miRNAs. The connections of protein-coding genes were inferred using co-expression analysis, and the interaction between miRNAs and their targets were predicted based on mismatch scores. The expression patterns of miRNAs and their predicted targets were visualized using heatmaps. The results indicated that the majority of target genes exhibited elevated expression levels in samples where the corresponding miRNAs were expressed at low levels (**Supplementary Figure S6**). The constructed regulatory network indicated that the regulators fall into two modules (**Figure 5**). Through analysis, we identified 24 TFs, 6 PKs, and 23 other genes (**Supplementary Table S9**) associated with the ABCDE gene (**Supplementary Table S10**). In the first module, two interacting genes, *PdFERR* and *PdAGL8a*, functioned as bub genes. *PdCOL11*, a CONSTANS-like gene that plays vital roles in the control of flowering time, was detected to be positively co-expressed with *PdFERR.* The transcripts of *PdCOL11* were predicted to be targeted by miRNAs of the miR164 family. A transcription factor *PdNAC071*, the target gene of miR159 and miR6445, were also positively correlated with *PdFERR* genes. Many genes of the ABCDE model were closely connected in the second model, such as *PdPI* and *PdAP3a/b/c* of the B-class and *PdSEP* genes and *PdAGL6* of the E-class. These genes were closely co-expressed with diverse transcription factors and protein kinases, including genes of GRF, MYB, and SERK families. Among the 71 protein-coding genes in the regulatory network, 35 genes were DMGs between female and male samples, indicating the significant roles of epigenetic modification in sex determination and flower development.

## 4 Discussion

### 4.1 Diverse regulatory genes for engineering of flower development

MADS-box genes play a critical role in various plant processes, including flower formation, regulation of flowering time, and flower development. In our analysis, we identified 96 MADS-box genes in *P. deltoides*, including 20 genes in the ABCDE model, the number are comparable to *P. trichocarpa* (Leseberg et al., 2006) and *Arabidopsis* (Parenicová et al., 2003). Among the genes in the ABCDE model, genes of B, C, and D classes exhibited tissue-specific expression, whereas genes of A and E classes are grouped into multiple clades with diverse expression patterns (**Figure 2B**). The large numbers of MADS-box genes encoded by the poplar genome and the diverse expression patterns of them ensure the fine-tunning of gene regulatory network to control the specification of organ identity, spatiotemporal regulation and the integration of hormonal and environmental signals (Castelán-Muñoz et al., 2019). The regulatory genes identified within the regulatory network may serve as potential candidates for engineering flower development.

Poplar “fluff,” which consists of seeds with cotton-like hairs, contributes significantly to air pollution. To mitigate the dispersal of poplar fluff, male poplar cultivars are preferred for cultivation. The sex-determining genes, such as *PtARR17* (Müller et al., 2020) in *P. tremula* and *PdFERR* in *P. deltoides* (Xue et al., 2020), can be utilized to design molecular markers that facilitate the screening of male individuals at the young seedling stage. Furthermore, the editing of *PtARR17* gene has been shown to convert female individuals into male counterparts (Müller et al., 2020), highlighting an opportunity to modify elite female cultivars. In MADS-box genes, B-class genes and C-class genes control the development of stamens and pistils respectively (Wan et al., 2025). The design of CRISPR guide RNAs targeting B-class and C-class genes, along with their co-regulated partners, could also be employed to generate environmentally friendly poplar trees that do not contribute to air pollution from fluff or pollen.

Manipulating genes that control reproductive phases represents another important strategy for modifying reproductive traits in poplar trees. A biallelic knockout of *Poplar STERILE APETALA* (*PopSAP*) results in complete reproductive sterility (Azeez and Busov, 2021). Additionally, knocking out *PopCEN1/PopCEN2* leads to early flowering by several months (Ortega et al., 2023). In *Arabidopsis, REM16* is reported to promote flowering through activation of the *SOC1-FT-AP1* pathway (Yu et al., 2020). A homologous gene corresponding to REM16 in *P. deltoides*, designated PdREM7, was also identified within our regulatory network. Exploring additional regulators may provide further insights into engineering reproductive traits in poplar species.

### 4.2 Correlations between sex determination genes and flower development genes

*PdFERR* in *P. deltoides*, *ARR17* in *P. tremula*, and their orthologous genes in other poplar species are sex-determining genes (Müller et al., 2020; Xue et al., 2020). *PdFERR* is highly expressed in female flower buds at the T3 stage (**Figure 2B**) and exhibits male-biased DNA methylation patterns in CG and CHG contexts. In the network analysis, *PdFERR* is co-expressed with diverse transcription factors, including *PdAGL8a*, *PdNAC029*, *PdMYB102*, *PdERF1B*, *PdHSFC1* and so on. The links between *PdFERR*/*PtARR17* and core genes in the ABCDE model provide clues for the downstream regulatory mechanisms of poplar sex-determining genes. A recent report indicated that an F-box transcriptional cofactor of *LFY*, the *UNUSUAL FLORAL ORGANS* (*UFO*) gene and the B-class gene *PISTILLATA* (*PI*) were de-repressed in the arr17 mutants (Leite Montalvão et al., 2022).

The correlation between *PdFERR* and other transcription factors suggests the interplay between *PdFERR* and multiple pathways. For example, CUC genes of the NAC family regulate the expansion of flower petals (Pei et al., 2013). Some CUC members are also identified as sex-determining genes in cucurbits (Pelayo and Wellmer, 2024). The interactions with the auxin-responsive gene (*PdSAUR4*) (Ren and Gray, 2015) and heat shock protein (*PdHSFC1*) (Scharf et al., 2012) suggest the regulation of *PdFERR* by hormone and environmental signals. A recent report also indicates the interaction between *PdFERR* and *ERF96* (Lu et al., 2023). Further characterization of gene interactions in the regulatory network would help to understand the labile sex expression in poplar (Mao et al., 2023).

### 4.3 Plasticity in sex determination and floral development

As a class of important regulatory molecules, 236 miRNAs were identified through small RNA sequencing. More than 4,500 genes, including eight MADS-box genes, were predicted as targets of these miRNAs, highlighting the role of post-transcriptional regulation in poplar flower development. Additionally, a significant number of genes exhibited differentially DNA methylated patterns between male and female flowers, particularly at stage T2. This observation underscores the importance of DNA methylation in maintaining flower differentiation and sex determination. The involvement of epigenetic modifications in flower development and sex determination may contribute to plasticity in sexual expression. A recent review indicated that among 22 angiosperm species studied, 18 (including *Populus*) have documented instances of labile sex expression (Käfer et al., 2022).

Sex conversions mediated by changes in DNA methylation levels have been observed across various species such as *Cucumis melo* (Martin et al., 2009; Latrasse et al., 2017), *D. kaki* (Akagi et al., 2016), *Sphaeropteris lepifera* (Zheng et al., 2023), *Silene pratensis* (Janoušek et al., 1996), and *Carica papaya* (Ávila-Hernández et al., 2023) both in natural settings and under controlled experimental conditions. Notably, the levels of methylation and transcription for sex-linked genes can vary between tissues, especially between female and male flowers, in monoecious plants like persimmon. This suggests that dynamic methylation modifications can precisely regulate gene expression (Akagi et al., 2016).

The instability induced by epigenetic modifications can enhance adaptability to environmental or nutritional conditions (Matsumoto et al., 2023). For instance, in cucumber (Lai et al., 2017) and *Ricinus communis* (Parvathy et al., 2021), temperature fluctuations during flower development stages can disrupt sex expression. This sexual lability may also play a crucial role in ensuring successful reproduction under circumstances of mate limitation, such as when individuals of one sex are scarce due to environmental stress (Käfer et al., 2017). In the case of *Mercurialis annua*, the removal of all males from populations resulted in leaky females, those capable of sporadically producing male flowers, exhibiting a significant increase in male flower production after four generations of natural selection (Cossard et al., 2021). The constructed regulatory network governing flower development in poplar, along with its regulation by DNA methylation and miRNAs, could provide insights into the dynamic regulation of flower development and potential labile sex expression.

## 5 Conclusions

In our analysis, we identified MADS-box genes differentially expressed during poplar flower development and constructed a regulatory network centered on these MADS-box genes. The correlations observed among the sex-determining gene *PdFERR*, miRNA genes, core genes in the ABCDE model, and other MADS-box regulators indicate complex regulatory interactions at multiple levels. The differential DNA methylation levels of the identified genes between female and male flower buds, along with the dynamics of DNA methylation throughout flower development, underscore regulation at the epigenetic level. The diverse interactions between sex-determining genes and MADS-boxes suggest variability in gene expression and sexual differentiation, particularly under changing environmental conditions. Our findings provide a set of candidate genes for further manipulation of flower development through transgenic approaches and genome editing techniques.

## Data Availability

The datasets presented in this study can be found in online repositories. The names of the repository/repositories and accession number(s) can be found in the article/**Supplementary Table S11**.
