# Supplementary material for: Transcriptional and epigenetic dynamics of sex determining gene PdFERR and MADS-box related genes during flower development in Populus deltoides
Source: Front Plant Sci. 2025 May 13;16:1582915. doi: 10.3389/fpls.2025.1582915 (PMC12106536; doi:10.3389/fpls.2025.1582915)
Supplement: Supplementary file 1 [file DataSheet1.docx]

Supplementary Material

# Supplementary Data

Supplementary Material should be uploaded separately on submission. Please include any supplementary data, figures and/or tables.

Supplementary material is not typeset so please ensure that all information is clearly presented, the appropriate caption is included in the file and not in the manuscript, and that the style conforms to the rest of the article.

# Supplementary Figures and Tables

## Supplementary Figures


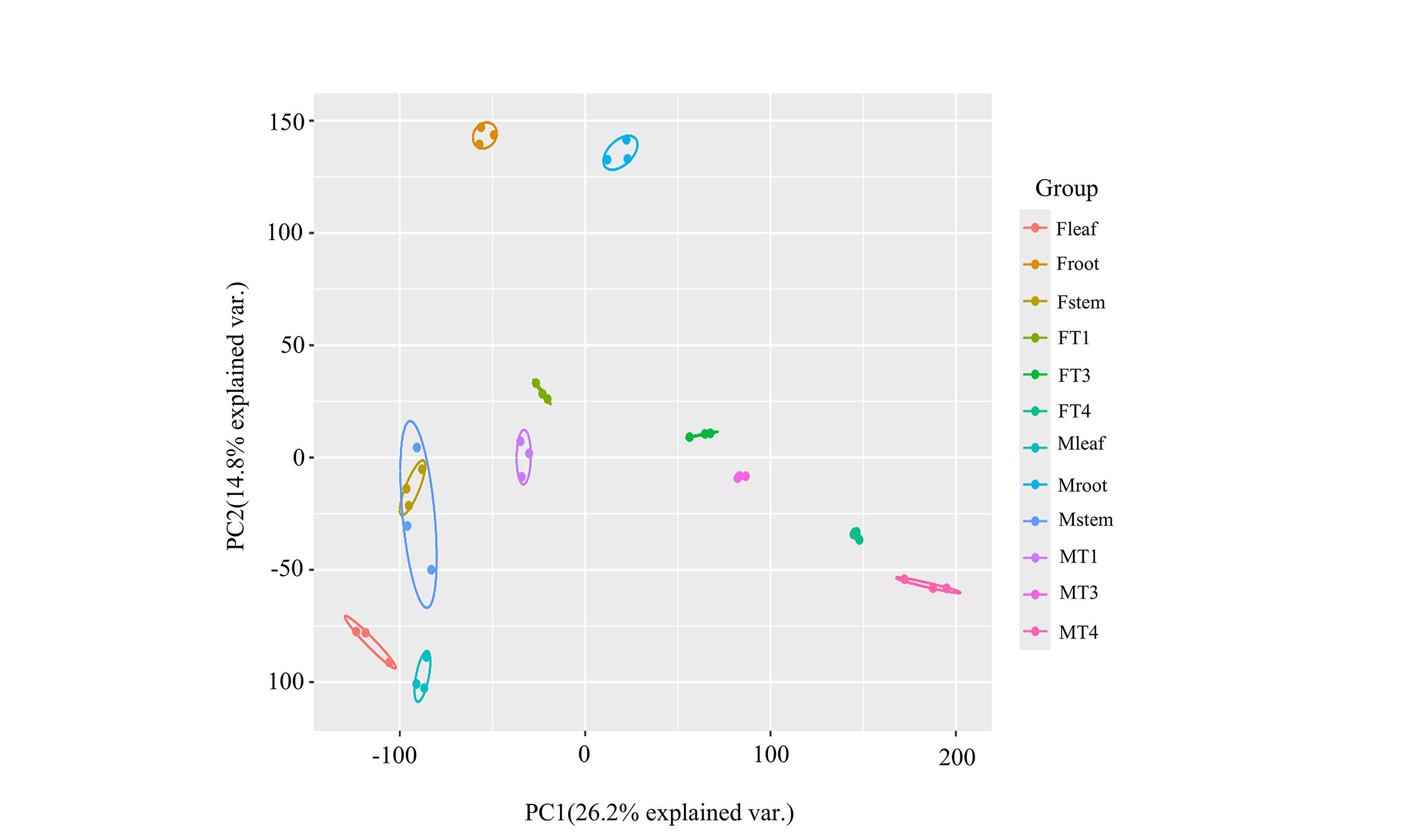


**Supplementary Figure 1.** Principal component analysis of flower buds and vegetative samples of *P. deltoides*.


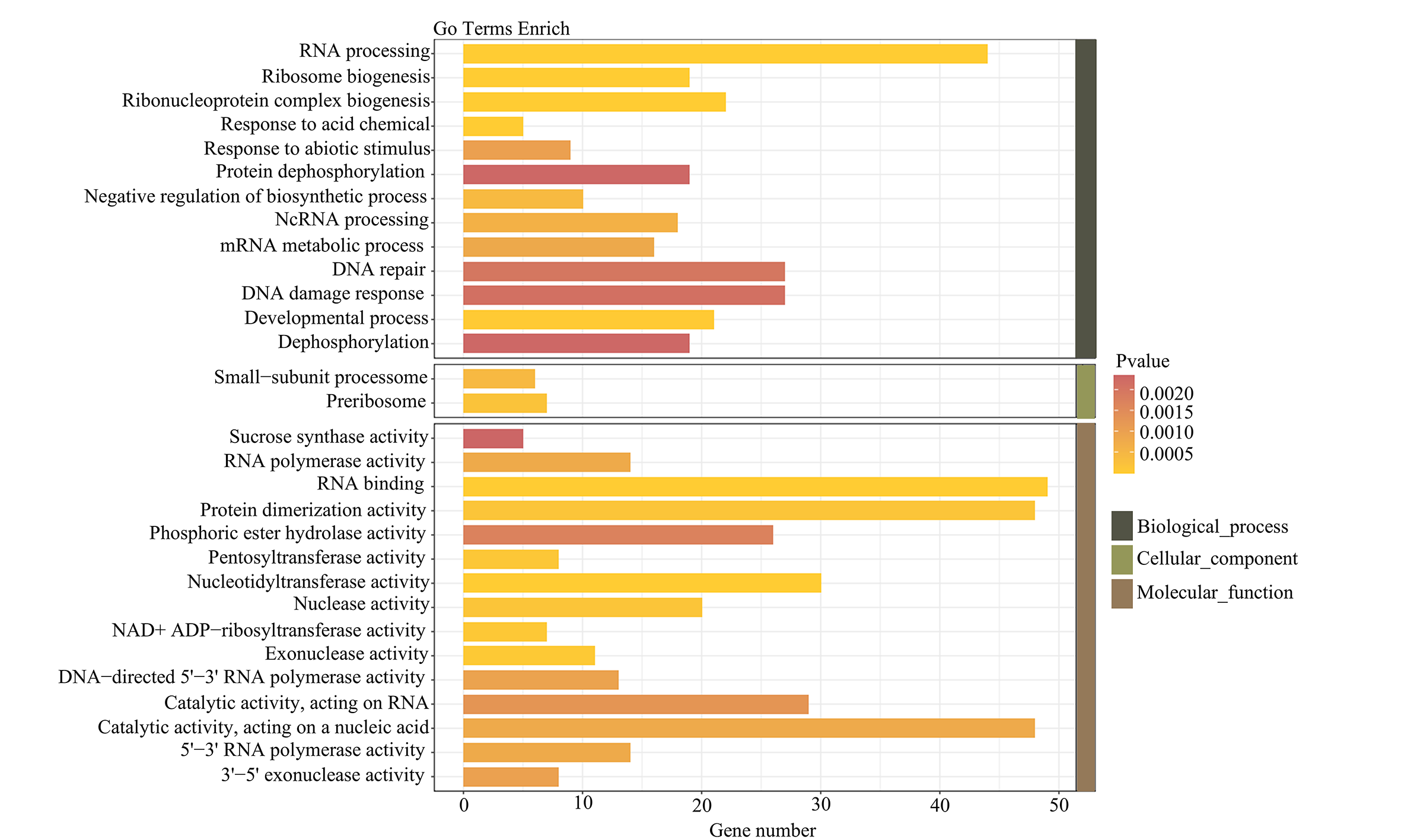


**Supplementary Figure 2.** GO enrichment analysis of genes predominantly expressed in both female and male flower buds.


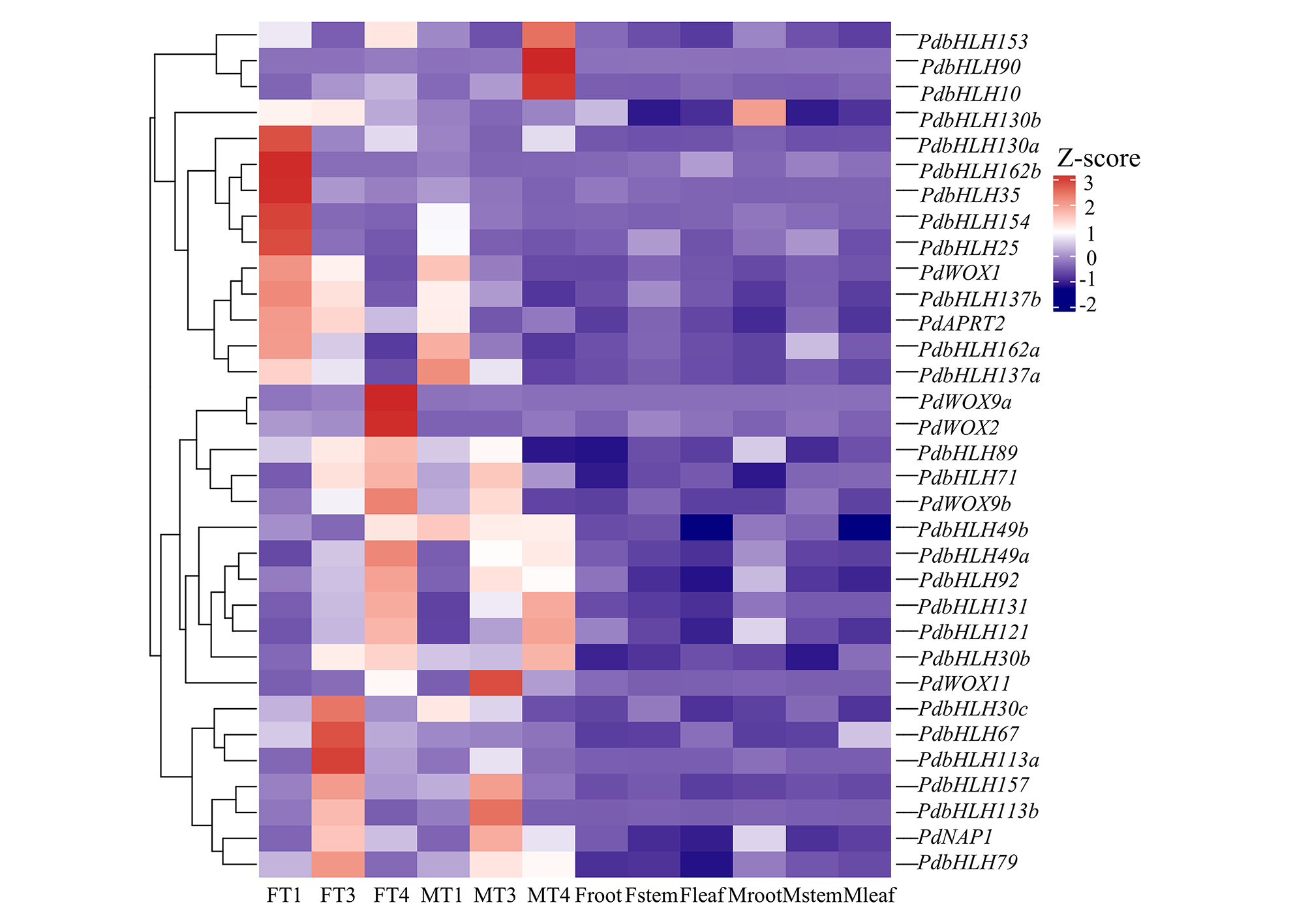


**Supplementary Figure 3.** **Expression patterns of genes in NAP, bHLH, and WOX gene families**. F and M indicate female and male plants, respectively. The expression values in flower buds and vegetative tissues, including root, stem, and leaf, were normalized by the gene for visualization.


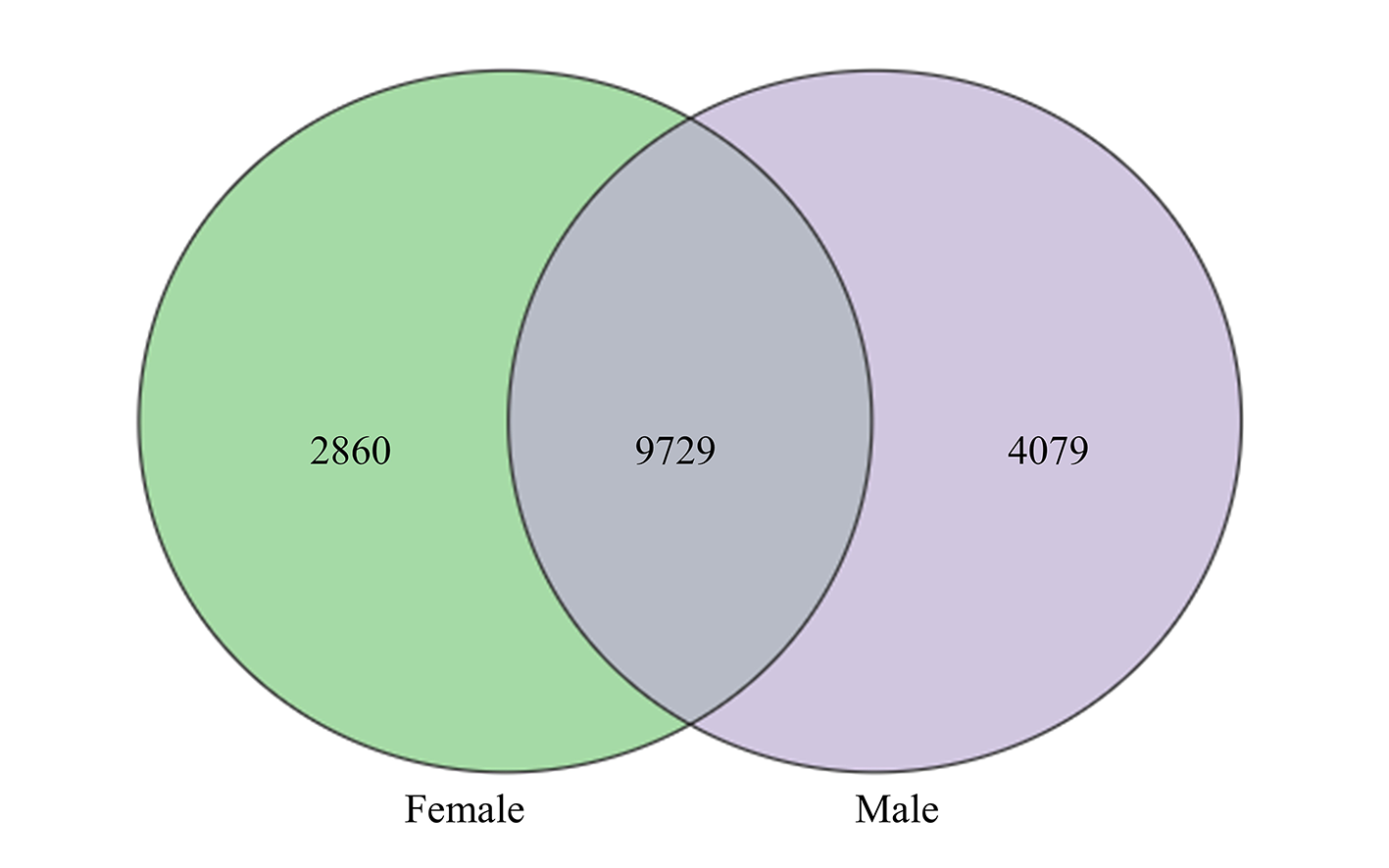


**Supplementary Figure 4.** **Venn diagrams of DEGs among developmental stages in female and male flower buds**. The DEGs were identified using ANOVA.


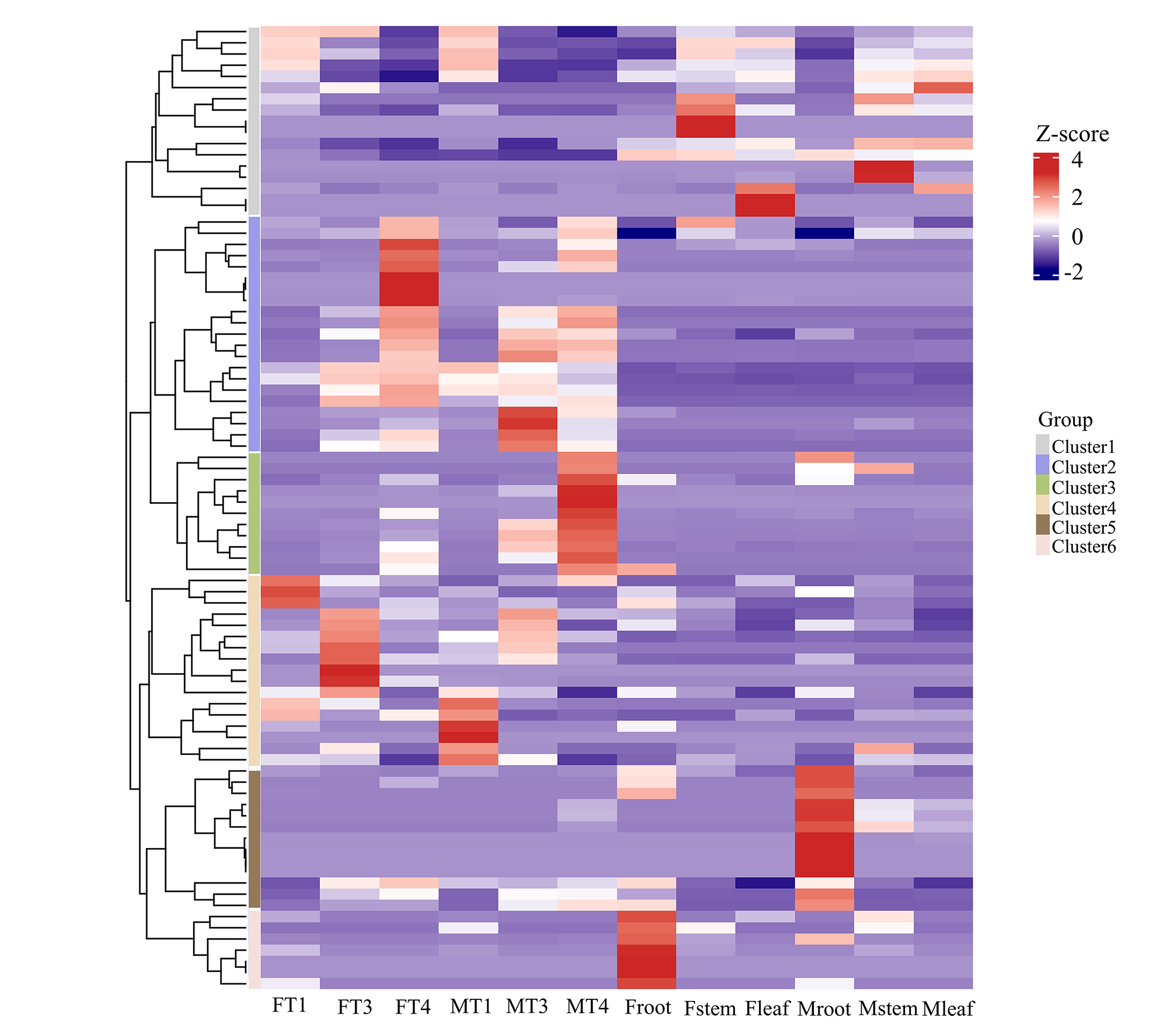


**Supplementary Figure 5.** **Expression patterns of genes in MADS-box gene family**. F and M indicate female and male plants, respectively. The expression values in flower buds and vegetative tissues, including root, stem, and leaf, were normalized by the gene for visualization


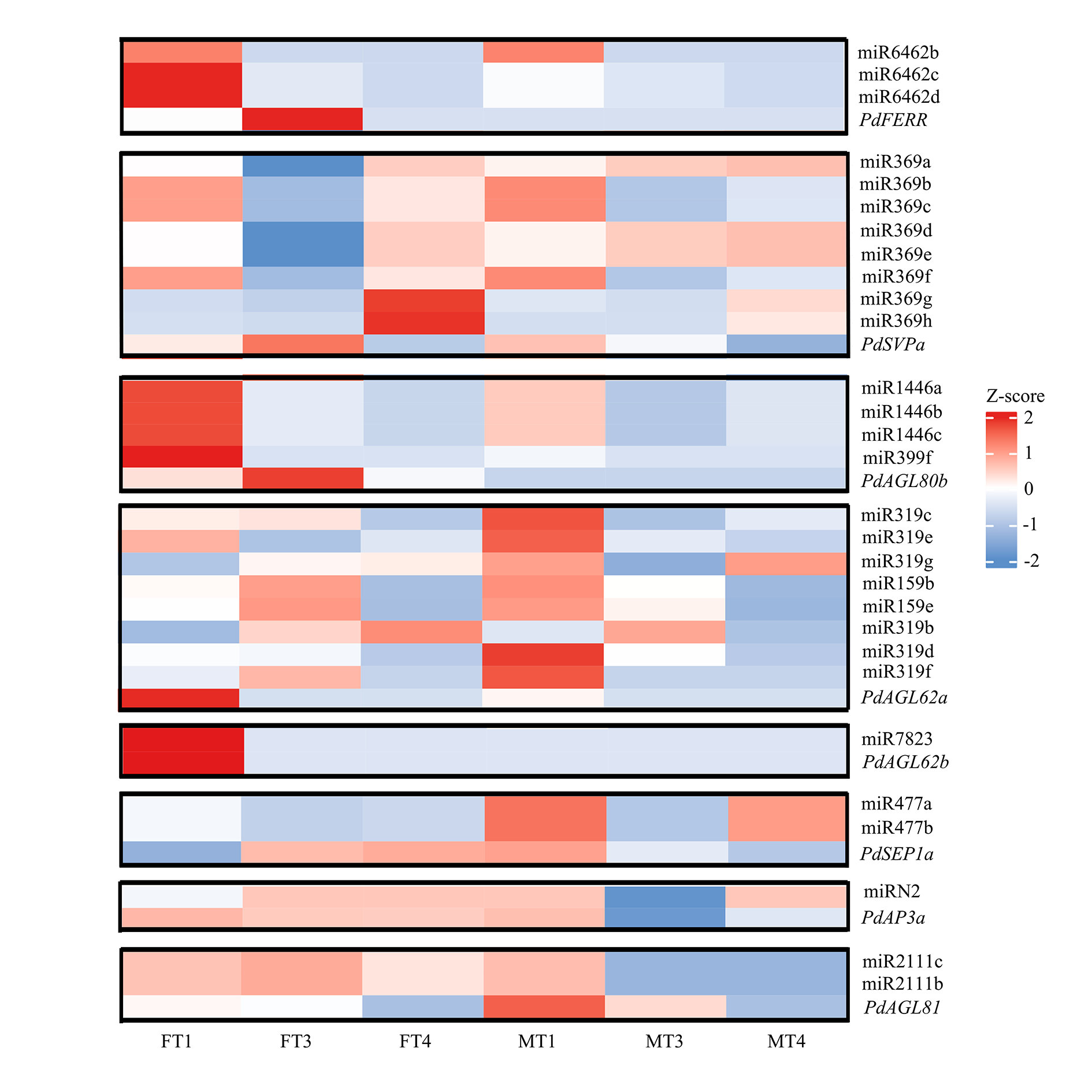


**Supplementary Figure 6.** **Heatmap of miRNA-mRNA expression.** F and M indicate female and male plants, respectively. The expression values in flower buds, were normalized by the gene for visualization. Each module represents the expression profiles of miRNAs and their corresponding target genes.

## Supplementary Tables

**Supplementary Table 1.** Sample list used in this study

| Sample classification | Sample tissue type | Sample ID | | Sampling date | Maximum  temperature | Minimum  temperature | Weather |
| --- | --- | --- | --- | --- | --- | --- | --- |
|  |  | Female | Male |  |  |  |  |
| Transcriptome data | Floral bud | FT1 | MT1 | 2018-06-18 | 31℃ | 23℃ | overcast |
|  |  | FT3 | MT3 | 2018-08-03 | 30℃ | 26℃ | moderate rain |
|  |  | FT4 | MT4 | 2018-12-01 | 18℃ | 12℃ | cloudy |
|  | Vegetative tissue | Froot | Mroot | 2018-06-18 | 31℃ | 23℃ | overcast |
|  |  | Fstem | Mstem | 2018-06-18 | 31℃ | 23℃ | overcast |
|  |  | Fleaf | Mleaf | 2018-06-18 | 31℃ | 23℃ | overcast |
| miRNA data | Floral bud | SFT1 | SMT1 | 2018-06-18 | 31℃ | 23℃ | overcast |
|  |  | SFT3 | SMT3 | 2018-08-03 | 30℃ | 26℃ | moderate rain |
|  |  | SFT4 | SMT4 | 2018-12-01 | 18℃ | 12℃ | cloudy |
| Methylation data | Floral bud | MFT2 | MMT2 | 2018-07-03 | 32℃ | 26℃ | cloudy |
|  |  | MFT4 | MMT4 | 2018-12-01 | 18℃ | 12℃ | cloudy |
|  |  | MFT5 | MMT5 | 2019-01-15 | 5℃ | -2℃ | cloudy |

**Supplementary Table 2.** Analysis groups of transcriptome, miRNA, and DNA methylation data.

| Analysis group | Comparison group |
| --- | --- |
| Analysis I | F others vs. F bud |
|  | M others vs. M bud |
| Analysis II | FT1、FT3、FT4 |
|  | MT1、MT3、MT4 |
| Analysis III | FT1 vs. MT1 |
|  | FT3 vs. MT3 |
|  | FT4 vs. MT4 |
| Analysis IV | SFT1 vs. SMT1 |
|  | SFT3 vs. SMT3 |
|  | SFT4 vs. SMT4 |
| Analysis V | MFT2 vs. MMT2 |
|  | MFT4 vs. MMT4 |
|  | MFT5 vs. MMT5 |

In comparison, the former samples are used as control

**Supplementary Table 3.** Gene identities of MADS-box genes except ABCDE genes.

| Gene ID | Name |  | Gene ID | Name |
| --- | --- | --- | --- | --- |
| *EVM0000518* | *PdSVPa* |  | *EVM0019487* | *PdAGL104a* |
| *EVM0000629* | *PdSVPb* |  | *EVM0020126* | *PdAGL24c* |
| *EVM0000968* | *PdAGL15* |  | *EVM0020342* | *PdAGL82* |
| *EVM0001845* | *PdMADS27* |  | *EVM0020402* | *PdAGL19a* |
| *EVM0002231* | *PdAGL12* |  | *EVM0020575* | *PdAGL62l* |
| *EVM0002396* | *PdAGL19* |  | *EVM0021171* | *PdAGL66* |
| *EVM0002681* | *PdMADS15* |  | *EVM0021695* | *PdAGL62m* |
| *EVM0002878* | *PdAGL80a* |  | *EVM0023212* | *PdMADS14* |
| *EVM0003319* | *PdAGL103a* |  | *EVM0023739* | *PdTT16* |
| *EVM0003343* | *PdAGL62d* |  | *EVM0024322* | *PdAGL104c* |
| *EVM0003605* | *PdAGL83* |  | *EVM0027579* | *PdAGL6c* |
| *EVM0004048* | *PdAGL80b* |  | *EVM0028059* | *PdAGL6d* |
| *EVM0004055* | *PdAGL62e* |  | *EVM0028537* | *PdAGL103c* |
| *EVM0004056* | *PdAGL62a* |  | *EVM0028916* | *PdAGL62n* |
| *EVM0005698* | *PdAGL12b* |  | *EVM0029418* | *PdAGL103b* |
| *EVM0006147* | *PdAGL62f* |  | *EVM0029515* | *PdAGL6e* |
| *EVM0006233* | *PdAGL6a* |  | *EVM0030050* | *PdAGL62c* |
| *EVM0006460* | *PdAGL62g* |  | *EVM0030789* | *PdAGL80c* |
| *EVM0006758* | *PdAGL80d* |  | *EVM0031437* | *PdAGL18* |
| *EVM0006909* | *PdSOC1a* |  | *EVM0031763* | *PdSOC1b* |
| *EVM0007094* | *PdAGL2* |  | *EVM0032323* | *PdAGL81* |
| *EVM0007267* | *PdAGL82a* |  | *EVM0032571* | *PdAGL82b* |
| *EVM0009700* | *PdAGL42* |  | *EVM0032845* | *PdAGL25* |
| *EVM0009769* | *PdSVPc* |  | *EVM0033032* | *PdMADS98* |
| *EVM0010039* | *PdSVPd* |  | *EVM0033242* | *PdAGL30b* |
| *EVM0010684* | *PdAGL27* |  | *EVM0033920* | *PdAGL65* |
| *EVM0010748* | *PdAGL24* |  | *EVM0034832* | *PdAGL19b* |
| *EVM0010958* | *PdMADS6* |  | *EVM0035219* | *PdAGL13* |
| *EVM0011407* | *PdAGL62b* |  | *EVM0035531* | *PdAGL16* |
| *EVM0011617* | *PdAGL62h* |  | *EVM0036185* | *PdAGL32b* |
| *EVM0012402* | *PdAGL32a* |  | *EVM0036414* | *PdAGL104d* |
| *EVM0012447* | *PdAGL62i* |  | *EVM0036648* | *PdAGL104e* |
| *EVM0013143* | *PdAGL80* |  | *EVM0037270* | *PdCAL* |
| *EVM0014521* | *PdMADS18* |  | *EVM0037892* | *PdAGL80e* |
| *EVM0015490* | *PdAGL30a* |  | *EVM0018093* | *PdAGL24a* |
| *EVM0015549* | *PdAGL104b* |  | *EVM0018412* | *PdAGL29* |
| *EVM0016458* | *PdAGL61* |  | *EVM0018541* | *PdAGL24b* |
| *EVM0017633* | *PdAGL62j* |  | *EVM0019112* | *PdAGL62k* |

**Supplementary Table 4.** Gene identities in the ABCDE model names of ABCDEs.

| Gene ID | Name |  | Gene ID | Name |
| --- | --- | --- | --- | --- |
| A genes | |  | D genes | |
| *EVM0017749* | *PdAP1a* |  | *EVM0010066* | *PdSHP1a* |
| *EVM0024267* | *PdAGL8a* |  | *EVM0030204* | *PdSHP1b* |
| *EVM0007089* | *PdAP1b* |  | *EVM0024562* | *PdSHP1c* |
| *EVM0012986* | *PdAP1c* |  | E genes | |
| *EVM0003892* | *PdAGL8b* |  | *EVM0024799* | *PdSEP3a* |
| B genes | |  | *EVM0014238* | *PdSEP1a* |
| *EVM0034894* | *PdPI* |  | *EVM0006458* | *PdSEP2* |
| *EVM0019205* | *PdAP3a* |  | *EVM0020105* | *PdAGL6b* |
| *EVM0002655* | *PdAP3b* |  | *EVM0037641* | *PdSEP1b* |
| *EVM0027701* | *PdAP3c* |  | *EVM0022762* | *PdSEP3b* |
| C genes | |  |  |  |
| *EVM0011726* | *PdAGL11a* |  |  |  |
| *EVM0001597* | *PdAGL11b* |  |  |  |

**Supplementary Table 7.** miRNAs target *PdFERR* and MADS-box genes.

| Name of miRNA | Target genes |  | Name of miRNA | Target genes |
| --- | --- | --- | --- | --- |
| miR6462b | *PdFERR* |  | miR319c | *PdAGL62a* |
| miR6462c | *PdFERR* |  | miR319e | *PdAGL62a* |
| miR6462d | *PdFERR* |  | miR319g | *PdAGL62a* |
| miR396a | *PdSVPa* |  | miR159b | *PdAGL62a* |
| miR396b | *PdSVPa* |  | miR159e | *PdAGL62a* |
| miR396c | *PdSVPa* |  | miR159d | *PdAGL62a* |
| miR396d | *PdSVPa* |  | miR319b | *PdAGL62a* |
| miR396e | *PdSVPa* |  | miR319d | *PdAGL62a* |
| miR396f | *PdSVPa* |  | miR319f | *PdAGL62a* |
| miR396g | *PdSVPa* |  | miR7823 | *PdAGL62b* |
| miR396h | *PdSVPa* |  | miR477a | *PdSEP1a* |
| miR1446a | *PdAGL80a* |  | miR477b | *PdSEP1a* |
| miR1446b | *PdAGL80a* |  | miRN2 | *PdAP3a* |
| miR1446c | *PdAGL80a* |  | miR2111b | *PdAGL81* |
| miR399f | *PdAGL80b* |  | miR2111c | *PdAGL81* |

**Supplementary Table 8.** MADS-box genes belong to DMGs.F and M indicate female and male plants respectively

| Gene ID | Name | T2 | | |  | T4 | | |  | | T5 | | |
| --- | --- | --- | --- | --- | --- | --- | --- | --- | --- | --- | --- | --- | --- |
|  |  | CG | CHG | CHH |  | CG | CHG | CHH | |  | CG | CHG | CHH |
| *EVM0030050* | *PdAGL62c* | F |  |  |  |  |  |  | |  |  |  |  |
| *EVM0021171* | *PdAGL66* | F | F |  |  |  |  |  | |  |  |  |  |
| *EVM0020402* | *PdAGL19a* | F |  |  |  |  |  |  | |  |  |  |  |
| *EVM0018412* | *PdAGL29* | F | F |  |  |  |  |  | |  |  |  |  |
| *EVM0011726* | *PdAGL11a* | F | F |  |  | M |  |  | |  |  |  |  |
| *EVM0003892* | *PdAGL8b* | F | F |  |  | M |  |  | |  |  |  | M |
| *EVM0000629* | *PdSVPb* | F |  |  |  |  |  |  | |  |  |  |  |
| *EVM0035531* | *PdAGL16* |  | F |  |  | F | F |  | |  | F | F |  |
| *EVM0037641* | *PdSEP1b* |  |  | F |  |  |  |  | |  |  |  |  |
| *EVM0032571* | *PdAGL82b* |  |  | F |  |  |  |  | |  |  |  |  |
| *EVM0004056* | *PdAGL62a* |  |  | F |  |  |  |  | |  |  |  |  |
| *EVM0029418* | *PdAGL103b* | F |  |  |  | M |  |  | |  |  |  |  |
| *EVM0032845* | *PdAGL25* |  |  |  |  |  |  |  | |  | F |  |  |
| *EVM0034832* | *PdAGL19b* | M | M |  |  |  |  |  | |  |  |  |  |
| *EVM0033032* | *PdMADS98* | M |  |  |  |  | M |  | |  | M |  |  |
| *EVM0032323* | *PdAGL81* | M |  |  |  |  |  |  | |  |  |  |  |
| *EVM0022762* | *PdSEP3b* | M |  |  |  |  |  |  | |  |  |  |  |
| *EVM0019487* | *PdAGL104a* | M |  |  |  |  |  |  | |  |  |  |  |
| *EVM0015490* | *PdAGL30a* | M |  |  |  |  |  |  | |  |  |  |  |
| *EVM0012402* | *PdAGL32a* | M |  |  |  |  |  |  | |  |  |  |  |
| *EVM0009769* | *PdSVPc* | M |  |  |  |  |  |  | |  |  |  |  |
| *EVM0006233* | *PdAGL6a* | M |  | M |  |  |  |  | |  |  |  |  |
| *EVM0001845* | *PdMADS27a* | M |  |  |  |  |  |  | |  |  |  |  |
| *EVM0030789* | *PdAGL80c* |  | M | M |  |  |  |  | |  |  |  |  |
| *EVM0010748* | *PdAGL24* |  | M |  |  |  |  |  | |  |  |  |  |
| *EVM0006758* | *PdAGL80d* |  | M |  |  |  |  |  | |  |  |  |  |
| *EVM0037892* | *PdAGL80e* |  |  | M |  |  |  |  | |  |  |  |  |
| *EVM0014521* | *PdMADS18* | M | M | M |  |  |  |  | |  |  |  |  |
| *EVM0009215* | *PdFERR* | M | M |  |  | M | M |  | |  | M | M | M |

F and M indicate female and male plants respectively.

**Supplementary Table 9.** Functional analysis of genes correlated with the ABCDE genes.

| Genes | Description | Action pathway |
| --- | --- | --- |
| *PdSAUR4* | SAUR-like auxin-responsive protein family | Hormone signal transduction |
| *PdILR1* | Hydrolyzes amino acid conjugates the plant growth regulator indole-3-acetic acid (IAA). Use Mg and Co ions as cofactors. | Hormone signal transduction |
| *PdAITR6* | ABA‐induced transcription repressor that acts as feedback regulator in ABA signaling. | Hormone signal transduction |
| *PdCUL1* | Involved in mediating responses to auxin and jasmonic acid. | Hormone signal transduction |
| *PdABI1* | Involved in abscisic acid (ABA) signal transduction. Negative regulator of ABA promotion of stomatal closure. | Hormone signal transduction |
| *PdGASA14* | Regulation of plant growth by GA-induced and DELLA-dependent signal transduction. | Hormone signal transduction |
| *PdEMH5* | It is activated by direct binding of *ABI5* to its promoter and is involved in the response to ABA. | Hormone signal transduction |
| *PdCYP707A4* | Involved in ABA catabolism | Hormone signal transduction |
| *PdPAT19* | Regulates BR signaling. | Hormone biosynthesis pathway |
| *PdXERICO* | Involved in ABA metabolism. | Hormone biosynthesis pathway |
| *PdPPRT1* | Putative C3HC4 zinc-finger ubiquitin E3 ligase, negative regulator in ABA and drought stress | Stress response |
| *PdLTI65* | Expression is induced under conditions of water deprivation (e.g. cold, high salt and dryness). | Stress response |
| *PdRCF3* | It is an upstream regulator of gene expression and heat tolerance in response to heat stress and a mediator of jasmonate signaling. | Stress response |
| *PdBUP1* | Involved in pollen tube development. | Signal pathway |
| *PdBUP2* | Involved in pollen tube development. | Signal pathway |
| *PdTIC* | Circadian gating of light responses. | Biological clock regulation pathway |
| *PdCER1* | Expression of the *CER1* gene associated with production of stem epicuticular wax and pollen fertility. | Biosynthesis of secondary metabolites |
| *PdGA2OX1* | *PdGA2OX1* expression is responsive to cytokinin and *KNOX* activities. | Diterpenoid biosynthesis |
| *PdFLP1* | Promotes early flowering independently of *FT*, induces the expression of SEPALLATA 3. | Chromatin remodeling |
|  |  |  |
| *PdMSH5* | Involved in meiotic recombination. | Meiotic recombination pathway |
| *PdSCI1* | Involved in auxin-dependent control of cell proliferation during pistil development. Loss-of-function mutations increase cell proliferation in the stigma. | Stem cell proliferation and differentiation pathway |
| *PdVIP2* | Elevation of *FLC* expression to a level capable of shaping the vernalisation response, the winter annual habit, to participate in floral control. | Transcriptional regulation |
| *PdRH35* | Spliceosomal component which gets ubiquinated and degraded through *COP1* under light-induced IR changes, modulating photomorphogenesis. | Hight signal transduction pathway |

**Supplementary Table 10.** Gene identities of genes associated with ABCDE genes.

| Gene ID | Name |  | Gene ID | Name |
| --- | --- | --- | --- | --- |
| *EVM0008963* | *PdSAUR4* |  | *EVM0009186* | *PdTIFY4B* |
| *EVM0032746* | *PdILR1* |  | *EVM0001376* | *PdRVE6* |
| *EVM0032723* | *PdAITR6* |  | *EVM0000148* | *PdGRF4a* |
| *EVM0008926* | *PdFLP1* |  | *EVM0007578* | *PdGRF4b* |
| *EVM0014038* | *PdCUL1* |  | *EVM0014119* | *PdREM7* |
| *EVM0012470* | *PdPPRT1* |  | *EVM0031340* | *PdMTERF1* |
| *EVM0035611* | *PdVIP2* |  | *EVM0025268* | *PdPIE1* |
| *EVM0030860* | *PdLTI65* |  | *EVM0032596* | *PdKNL2* |
| *EVM0007216* | *PdABI1* |  | *EVM0019740* | *PdCIPK24* |
| *EVM0032411* | *PdGASA14* |  | *EVM0021272* | *PdRLT1* |
| *EVM0034144* | *PdEMH5* |  | *EVM0017338* | *PdGRF4c* |
| *EVM0031551* | *PdCYP707A4* |  | *EVM0026118* | *PdSERK1* |
| *EVM0031313* | *PdTIC* |  | *EVM0033872* | *PdPERK1* |
| *EVM0035578* | *PdMADS27b* |  | *EVM0009605* | *PdPHL6* |
| *EVM0007475* | *PdCER1* |  | *EVM0002391* | *PdPERK8b* |
| *EVM0017513* | *PdXERICO* |  | *EVM0006159* | *PdNFP* |
| *EVM0015841* | *PdGA2OX1* |  | *EVM0000628* | *PdMYB102* |
| *EVM0005728* | *PdbHLH145* |  | *EVM0001267* | *PdNAC071* |
| *EVM0025999* | *PdBUP1* |  | *EVM0029024* | *PdCOL11* |
| *EVM0001913* | *PdBUP2* |  | *EVM0029609* | *PdbHLH67* |
| *EVM0022163* | *PdMSH5* |  | *EVM0005136* | *PdNAC029* |
| *EVM0023402* | *PdSCI1* |  | *EVM0006128* | *PdKUA1* |
| *EVM0011823* | *PdRCF3* |  | *EVM0007185* | *PdHSFC1* |
| *EVM0013948* | *PdRH35* |  | *EVM0018092* | *PdATC3H20* |
| *EVM0030580* | *PdPAT19* |  | *EVM0034929* | *PdERF1B* |
| *EVM0014717* | *PdNDX* |  | *EVM0033837* | *PdGNAT8* |
| *EVM0009611* | *PdPERK8a* |  |  |  |

**Supplementary Table 11.** NCBI SRA accession numbers for all sequencing runs.

| Assay Type | Run | BioSample | Experiment | Stage | Sample Name |
| --- | --- | --- | --- | --- | --- |
| RNA-Seq | SRR11635789 | SAMN14764841 | SRX8199898 | T1 | FT1_1 |
| RNA-Seq | SRR11635788 | SAMN14764842 | SRX8199899 | T1 | FT1_2 |
| RNA-Seq | SRR11635797 | SAMN14764843 | SRX8199890 | T1 | FT1_3 |
| RNA-Seq | SRR11635796 | SAMN14764844 | SRX8199891 | T3 | FT3_1 |
| RNA-Seq | SRR11635795 | SAMN14764845 | SRX8199892 | T3 | FT3_2 |
| RNA-Seq | SRR11635794 | SAMN14764846 | SRX8199893 | T3 | FT3_3 |
| RNA-Seq | SRR11635793 | SAMN14764847 | SRX8199894 | T4 | FT4_1 |
| RNA-Seq | SRR11635792 | SAMN14764848 | SRX8199895 | T4 | FT4_2 |
| RNA-Seq | SRR11635791 | SAMN14764849 | SRX8199896 | T4 | FT4_3 |
| RNA-Seq | SRR11635790 | SAMN14764850 | SRX8199897 | T1 | MT1_1 |
| RNA-Seq | SRR11635787 | SAMN14764851 | SRX8199900 | T1 | MT1_2 |
| RNA-Seq | SRR11635786 | SAMN14764852 | SRX8199901 | T1 | MT1_3 |
| RNA-Seq | SRR11635803 | SAMN14764853 | SRX8199884 | T3 | MT3_1 |
| RNA-Seq | SRR11635802 | SAMN14764854 | SRX8199885 | T3 | MT3_2 |
| RNA-Seq | SRR11635801 | SAMN14764855 | SRX8199886 | T3 | MT3_3 |
| RNA-Seq | SRR11635800 | SAMN14764856 | SRX8199887 | T4 | MT4_1 |
| RNA-Seq | SRR11635798 | SAMN14764858 | SRX8199889 | T4 | MT4_3 |
| RNA-Seq | SRR11635799 | SAMN14764857 | SRX8199888 | T4 | MT4_2 |
| Bisulfite-Seq | SRR11635555 | SAMN14764527 | SRX8199670 | T2 | MFT2_1 |
| Bisulfite-Seq | SRR11635554 | SAMN14764528 | SRX8199671 | T2 | MFT2_2 |
| Bisulfite-Seq | SRR11635563 | SAMN14764529 | SRX8199662 | T2 | MFT2_3 |
| Bisulfite-Seq | SRR11635562 | SAMN14764530 | SRX8199663 | T4 | MFT4_1 |
| Bisulfite-Seq | SRR11635561 | SAMN14764531 | SRX8199664 | T4 | MFT4_2 |
| Bisulfite-Seq | SRR11635560 | SAMN14764532 | SRX8199665 | T4 | MFT4_3 |
| Bisulfite-Seq | SRR11635559 | SAMN14764533 | SRX8199666 | T5 | MFT5_1 |
| Bisulfite-Seq | SRR11635558 | SAMN14764534 | SRX8199667 | T5 | MFT5_2 |
| Bisulfite-Seq | SRR11635557 | SAMN14764535 | SRX8199668 | T5 | MFT5_3 |
| Bisulfite-Seq | SRR11635556 | SAMN14764536 | SRX8199669 | T2 | MMT2_1 |
| Bisulfite-Seq | SRR11635553 | SAMN14764537 | SRX8199672 | T2 | MMT2_2 |
| Bisulfite-Seq | SRR11635552 | SAMN14764538 | SRX8199673 | T2 | MMT2_3 |
| Bisulfite-Seq | SRR11635569 | SAMN14764539 | SRX8199656 | T4 | MMT4_1 |
| Bisulfite-Seq | SRR11635568 | SAMN14764540 | SRX8199657 | T4 | MMT4_2 |
| Bisulfite-Seq | SRR11635567 | SAMN14764541 | SRX8199658 | T4 | MMT4_3 |
| Bisulfite-Seq | SRR11635566 | SAMN14764542 | SRX8199659 | T5 | MMT5_1 |
| Bisulfite-Seq | SRR11635565 | SAMN14764543 | SRX8199660 | T5 | MMT5_2 |
| Bisulfite-Seq | SRR11635564 | SAMN14764544 | SRX8199661 | T5 | MMT5_3 |
| miRNA-Seq | SRR11625373 | SAMN14755252 | SRX8189465 | T1 | SFT1_1 |
| miRNA-Seq | SRR11625372 | SAMN14755253 | SRX8189466 | T1 | SFT1_2 |
| miRNA-Seq | SRR11625363 | SAMN14755254 | SRX8189475 | T1 | SFT1_3 |
| miRNA-Seq | SRR11625362 | SAMN14755255 | SRX8189476 | T3 | SFT3_1 |
| miRNA-Seq | SRR11625361 | SAMN14755256 | SRX8189477 | T3 | SFT3_2 |
| miRNA-Seq | SRR11625360 | SAMN14755257 | SRX8189478 | T3 | SFT3_3 |
| miRNA-Seq | SRR11625359 | SAMN14755258 | SRX8189479 | T4 | SFT4_1 |
| miRNA-Seq | SRR11625358 | SAMN14755259 | SRX8189480 | T4 | SFT4_2 |
| miRNA-Seq | SRR11625357 | SAMN14755260 | SRX8189481 | T4 | SFT4_3 |
| miRNA-Seq | SRR11625356 | SAMN14755261 | SRX8189482 | T1 | SMT1_1 |
| miRNA-Seq | SRR11625371 | SAMN14755262 | SRX8189467 | T1 | SMT1_2 |
| miRNA-Seq | SRR11625370 | SAMN14755263 | SRX8189468 | T1 | SMT1_3 |
| miRNA-Seq | SRR11625369 | SAMN14755264 | SRX8189469 | T3 | SMT3_1 |
| miRNA-Seq | SRR11625368 | SAMN14755265 | SRX8189470 | T3 | SMT3_2 |
| miRNA-Seq | SRR11625367 | SAMN14755266 | SRX8189471 | T3 | SMT3_3 |
| miRNA-Seq | SRR11625366 | SAMN14755267 | SRX8189472 | T4 | SMT4_1 |
| miRNA-Seq | SRR11625365 | SAMN14755268 | SRX8189473 | T4 | SMT4_2 |
| miRNA-Seq | SRR11625364 | SAMN14755269 | SRX8189474 | T4 | SMT4_3 |
